# Primary Epithelioid Angiosarcoma of the Tibia: A Case Report and Review of the Literature

**DOI:** 10.7759/cureus.70208

**Published:** 2024-09-25

**Authors:** Ryuta Iwanaga, Atsushi Mihara, Keiichi Muramatsu, Takashi Sakai

**Affiliations:** 1 Department of Orthopedic Surgery, Yamaguchi University Graduate School of Medicine, Ube, JPN; 2 Department of Hand and Microsurgery, Nagato General Hospital, Nagato, JPN

**Keywords:** amputation, bone tumors, epithelioid angiosarcoma, rare cancers, tibia

## Abstract

Angiosarcoma of the bone is very rare, accounting for less than 1% of all malignant bone tumors. We report our experience with an epithelioid hemangiosarcoma arising in the proximal tibia and a review of the literature. The patient, an 85-year-old male, was referred to our institution because of left knee pain that had persisted for five months, and bone radiolucency was observed in the proximal tibia. A bone and prostate biopsy was performed due to a suspicion of prostate cancer and bone metastasis. The positron emission tomography-computed tomography (PET-CT) showed accumulation in the prostate and proximal tibia, and the prostate-specific antigen (PSA) level was high at 14.11 ng/mL. Therefore, we diagnosed the patient with bone metastasis of prostate cancer and performed curettage and cement filling. However, postoperative pathological diagnosis revealed an epithelioid hemangiosarcoma, and we considered amputation. Two months after curettage, the patient underwent transfemoral amputation because of local recurrence. Eight months after amputation, he died due to multiple metastases. Approximately 20% of cases with epithelioid hemangiosarcoma have multiple metastases at the time of initial diagnosis, and it is sometimes difficult to distinguish from bone metastases of cancer because they may be arranged in foci or on cords. There are few reports of effective adjuvant therapy, and the clinical course can be rapid, so early amputation should be considered.

## Introduction

Angiosarcomas mostly occur in skin and soft tissues and account for 2% of malignant soft tissue tumors. They have been reported in the breast, liver, spleen, heart, and bone. Primary angiosarcoma of the bone is very rare and accounts for <1% of malignant bone tumors [1].

Epithelioid angiosarcoma shows epithelioid morphology and may be misdiagnosed as bone metastases in pathological diagnosis. It can also be misdiagnosed clinically as bone metastasis of cancer, since it occurs in solitary or multiple locations [2], mainly in the lower extremities. Accurate diagnosis of this tumor is usually delayed, and treatment options are limited, resulting in generally poor outcomes [3].

Here, we present a very rare case of angiosarcoma arising from the proximal tibia and discuss the differential diagnosis and appropriate treatment for this difficult lesion.

## Case presentation

An 85-year-old male presented with a history of pain and swelling in the left knee for five months duration. He had tenderness in the proximal tibia region, and the pain worsened with walking. No abnormalities were detected in the laboratory tests, except for increased alkaline phosphatase and prostate-specific antigen (PSA). Laboratory results are as follows: for complete blood count (CBC), white blood cell of 5,920×10^6^/L, hemoglobin of 131 g/L, hematocrit of 38.9%, platelet of 16×10^9^/L, prothrombin time (PT) of 11.5 seconds, normal, and PT/international normalized ratio (INR) of 0.99; for serum chemistries, sodium of 137 mmol/L, potassium of 4.4 mmol/L, chloride of 99 mmol/L, blood urea nitrogen (BUN) of 15 mg/dL, creatinine of 0.56 mg/dL, glucose of 111 mg/dL, total protein of 7.9 g/dL, and albumin of 5 g/dL. In addition, the total bilirubin was 0.5 mg/dL, aspartate aminotransferase (AST) was 22 U/L, alanine transaminase (ALT) was 16 U/L, and lactate dehydrogenase was 189 U/L. Furthermore, alkaline phosphatase was 466 U/L, which was slightly elevated. For tumor markers, cancer antigen 125 (CA125) was 9 U/mL, CA15-3 was 13.7 U/mL, squamous cell carcinoma antigen (SCC) was 0.5 ng/mL, and CA19-9 was 7.6 U/mL. Also, PSA is 14.11 ng/mL, which is slightly elevated.

**Figure 1:**
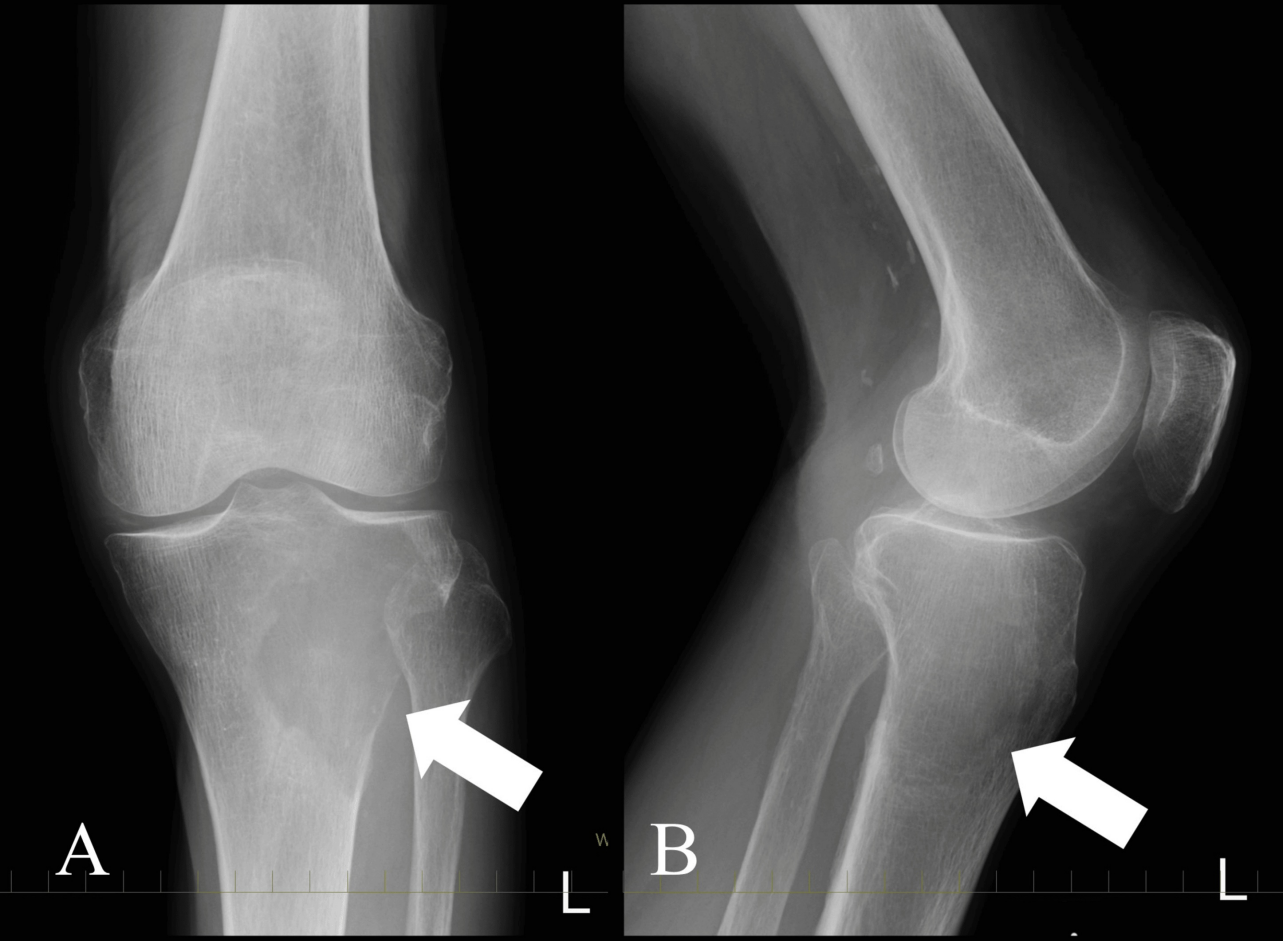
Radiograph at the initial visit Imaging findings of the knee of an 86-year-old male affected with an epithelioid angiosarcoma of the proximal tibia. A and B: The radiographs show significant bone destruction in the medial proximal tibia (white arrows). There is no periosteal reaction, no osteosclerosis of the tumor margins, and no calcification within the tumor.

Plain radiographs showed an osteolytic lesion with undefined margins and destruction of cortical and medullary bone. A large soft tissue mass was apparent in the proximal epiphysis of the left tibia. Neither periosteal reaction nor calcification was observed in the lesion (Figure 1).

**Figure 2:**
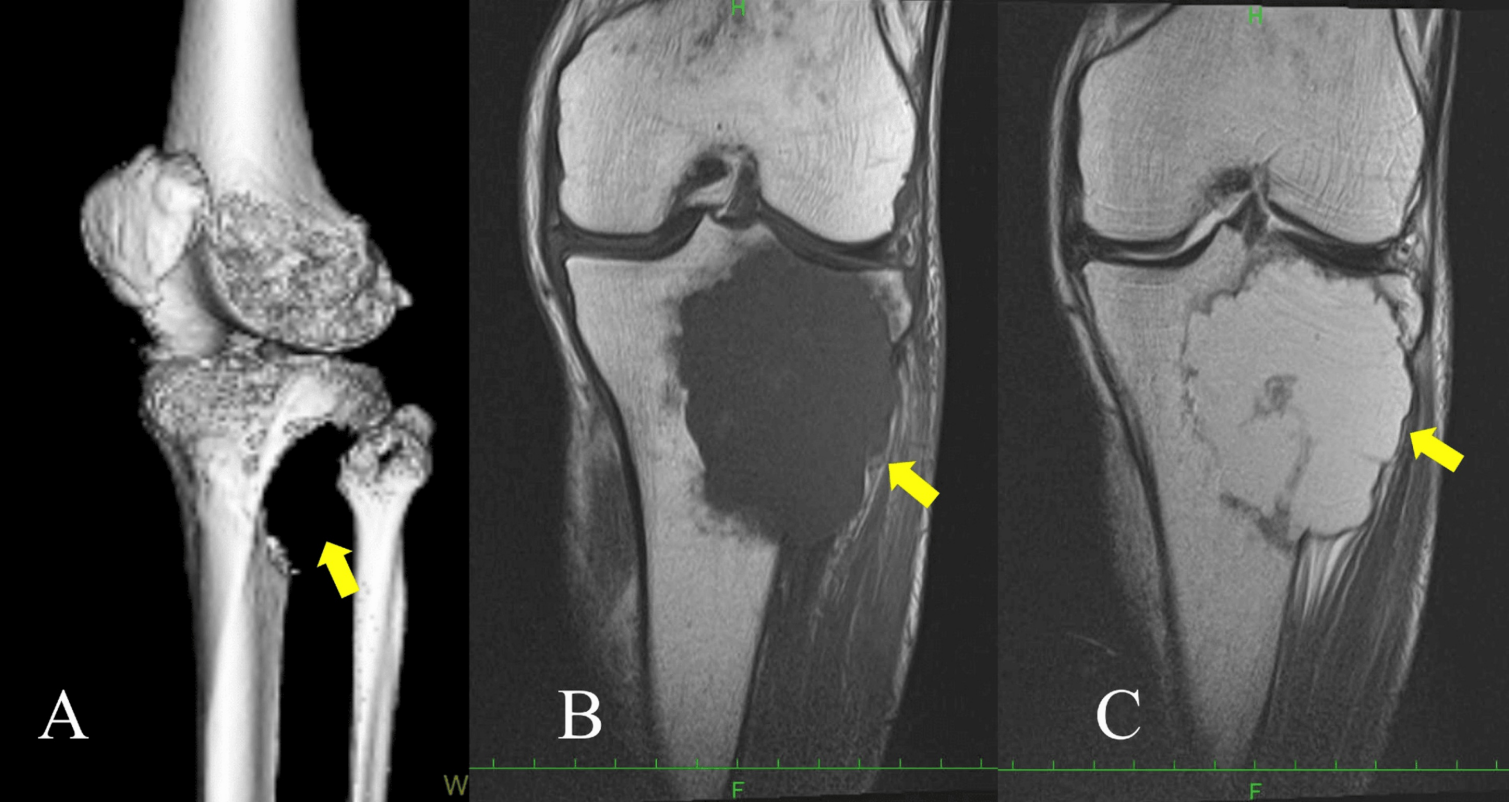
CT and MRI CT and MRI findings of the knee of an 86-year-old male affected with an epithelioid angiosarcoma of the proximal tibia. A: The cortical bone of the proximal medial tibia has disappeared as in Figure 1 (3DCT). B: The tumor shows a low signal (T1-weighted image, coronal plane). C: The tumor is a high signal, partially low signal, and heterogeneous (T2-weighted image). In both images, the tumor has destroyed the cortical bone of the proximal tibia and invaded the soft tissue (yellow arrows). CT: computed tomography, MRI: magnetic resonance imaging, 3DCT: three-dimensional computed tomography

Computed tomography (CT) showed that the cortical bone of the proximal tibia was destroyed on the posterior and lateral sides. On magnetic resonance imaging (MRI), the tumor was detected as a destructive lesion occupying the proximal tibia. The lesion displayed low signal intensity on T1-weighted images and heterogeneous signal intensity on T2-weighted images (Figure 2).

**Figure 3:**
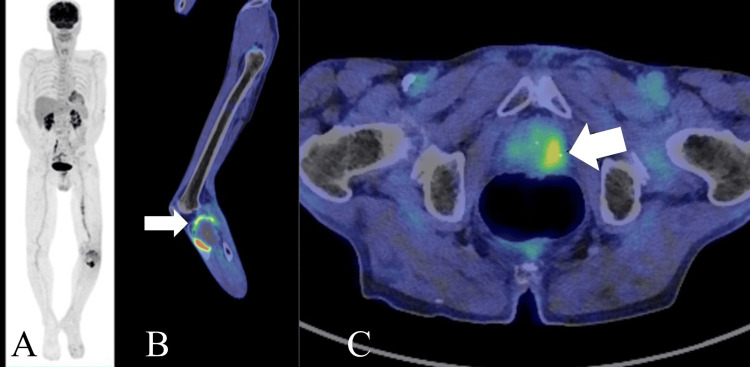
FDG-PET FDG-PET findings of the knee of an 86-year-old male affected with an epithelioid angiosarcoma of the proximal tibia. A: Overall view. B: SUVmax 7 uptake in the left tibial tumor (white arrow). C: SUVmax 4 uptake in the left prostate (white arrow). FDG-PET: (18F)-2-fluoro-2-deoxy-D-glucose-positron emission tomography, SUVmax: maximum standardized uptake value

On (18F)-2-fluoro-2-deoxy-D-glucose-positron emission tomography (FDG-PET), the tumor in the tibia and prostate showed up as areas of increased uptake (Figure 3). Multiple tumor markers including α-fetoprotein, carcinoembryonic antigen, CA125, CA19-9, interleukin-2, and squamous cell carcinoma antigen were examined. All were found to show normal levels, except for an increase in prostate-specific antigen (PSA) (14.11 ng/mL). At this point, we suspected metastatic prostatic carcinoma due to the high PSA level and the PET findings.

**Figure 4:**
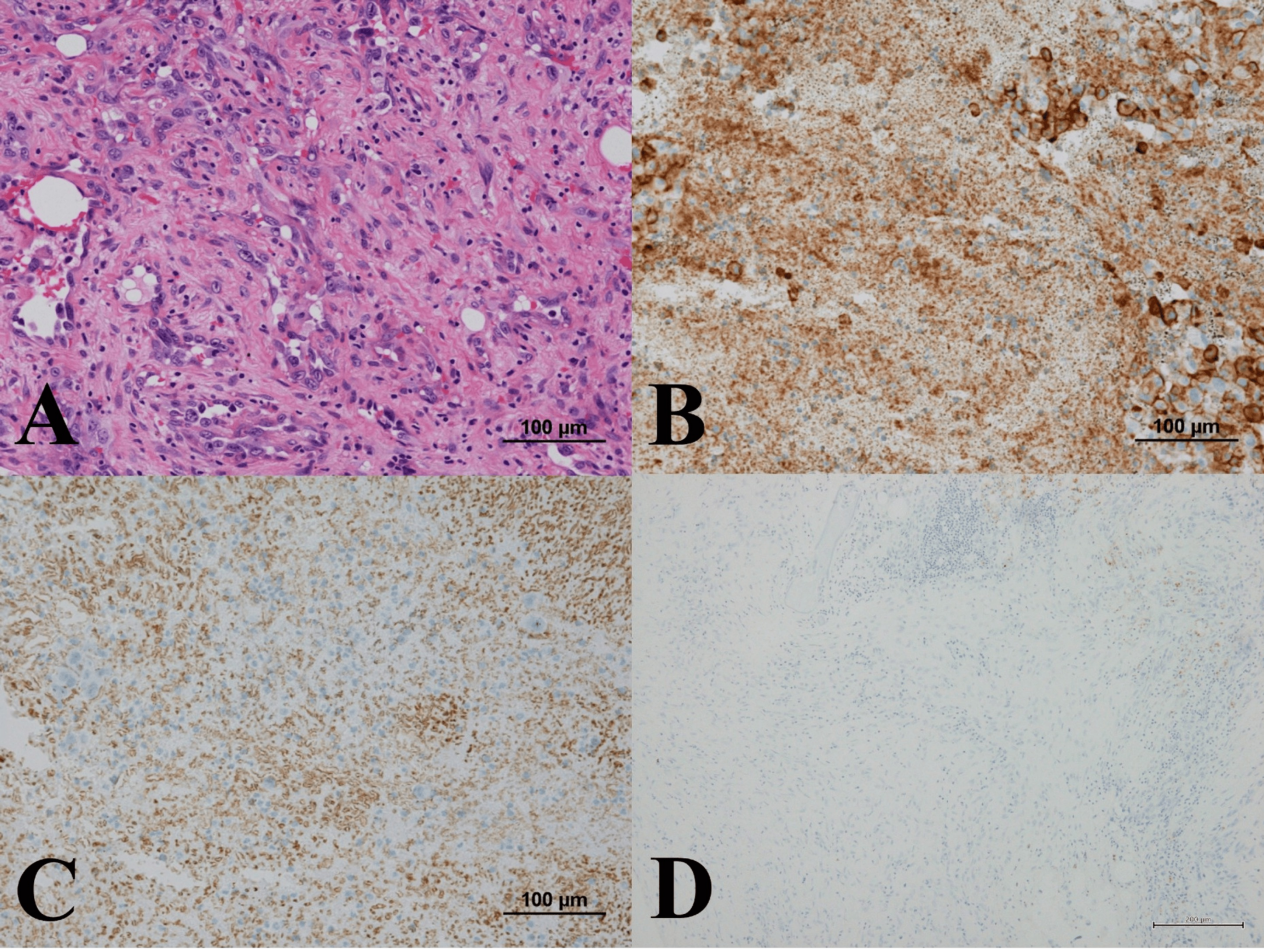
Microscopic appearance of the specimen from the tumor Histological diagnosis was made from the curettage tumor tissue. A: Hematoxylin and eosin stain (×200 magnification): nuclear swelling and irregular-sized, highly atypical tumor cells with epithelial-like arrangement and proliferation. B: CD31, positive. C: CD34, positive. D: PSA, negative. PSA: prostate-specific antigen

A histological specimen obtained from needle biopsy suggested a diagnosis of metastatic carcinoma due to the epithelial morphology. A diagnosis of bone metastasis from prostate cancer was made, and curettage with augmentation by poly(methyl methacrylate) (PMMA) was performed. After the curettage, the diagnosis was revised on account of the histological findings of epithelioid angiosarcoma. Hematoxylin and eosin-stained sections revealed sheets and nests of large, pleomorphic epithelioid cells with a vesicular or epithelioid shape pattern (Figure 4A).

Immunohistochemically, it showed that the tumor cells were positive for vimentin, CD31 (Figure 4B), cytokeratin AE1/AE3, cytokeratin 7, and CD34 (Figure 4C) but negative for PSA (Figure 4D). These findings are consistent with a diagnosis of epithelioid angiosarcoma.

**Figure 5:**
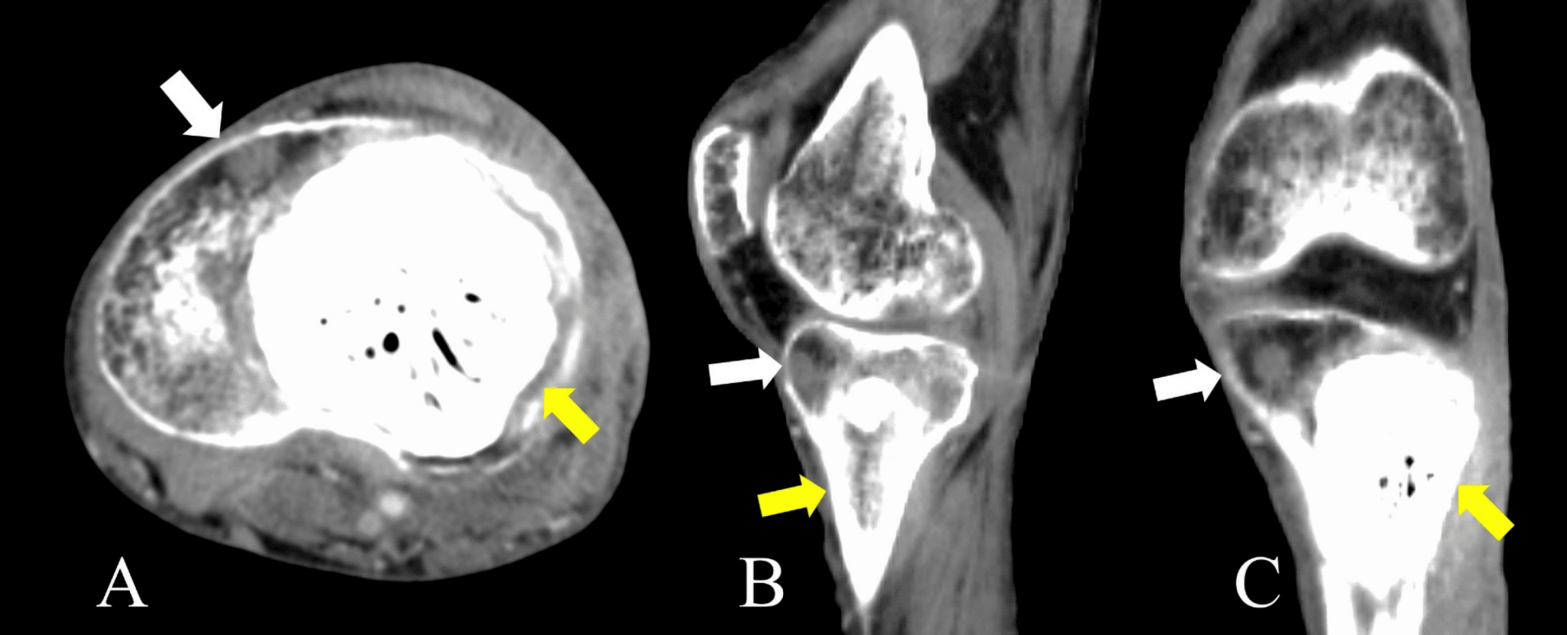
Enhanced CT two months after curettage A: Axial plane. B: Sagittal plane. C: Coronal plane. The curettage area is filled with PMMA (yellow arrows). There is an enhanced lesion at the edge of the curetted tumor that is thought to be a recurrent lesion (white arrows). CT: computed tomography, PMMA: poly(methyl methacrylate)

The patient continued to bleed from the wound for seven weeks. We tried to stop the bleeding by using intravenous hemostatic agents; however, the bleeding did not stop. Seven weeks after curettage, CT showed local recurrence in the proximal tibia (Figure 5).

Two months after curettage, we performed an above-the-knee amputation. Specimens from the amputated leg showed a hemorrhagic tumor mass invading the upper end of the tibia with erosion of the cortex. The amputated thigh healed without bleeding, and there was no local recurrence. However, eight months after amputation, he died due to multiple metastases.

## Discussion

Diagnosis of angiosarcoma of the bone

Obtaining an accurate diagnosis of angiosarcoma arising from bone is extremely difficult. Angiosarcoma of the bone most commonly occurs in the femur and tibia, followed by the pelvis, vertebrae, and bones of the upper extremities [4]. The radiographic features of angiosarcomas of the bone are not specific. Osteolytic lesions with indistinct borders are the predominant feature, and these commonly involve adjacent soft tissues.

In histopathologic diagnosis, angiosarcoma can be difficult to differentiate from epithelioid hemangioendothelioma. The latter is an intermediate-grade malignant hemangioma with similar radiographic findings and clinical presentation. However, in contrast to angiosarcoma, epithelioid hemangioendothelioma rarely causes cortical destruction or invasion of soft tissues [5]. In our case, there was posterior and lateral cortical destruction of the left tibia on CT and soft tissue invasion on MRI. Metastatic carcinoma can also be difficult to differentiate from angiosarcoma. Both tumors are composed of epithelioid neoplastic cells and tend to affect the elderly. Another misdiagnosis is multifocal lesion [6]. In a previous report, six of 10 cases were multifocal and three of 10 cases were misdiagnosed as metastatic cancer [7].

Therefore, it is beneficial to perform a histological examination after biopsy of the bone lesion. However, if a complete epithelial-like collection is observed in the needle biopsy specimen, it is likely to lead to a false inaccurate diagnosis. The accuracy of fine needle aspiration cytology is estimated to be 87.8% [8]. Histological features of epithelioid angiosarcoma include the presence of well-formed vascular vessels and mucin-negative cytoplasmic vacuoles; it may also contain fragmented erythrocytes within them. There is also an intratumoral neutrophil infiltrate, as well as the expression of vascular markers by immunohistochemistry [7]. Angiosarcoma of the bone is immunopositive for epithelial markers such as keratin, cytokeratin, and high-molecular-weight keratin. Angiosarcoma of the bone is also immunopositive for vascular endothelial markers such as CD31, CD34, von Willebrand factor (vWf), and factor VIII-related antigen. The majority of angiosarcomas are positive for CD31 expression [9,10]. In our case, CD31, CD34, and cytokeratin expressions were all positive, while both factor VIII and vWf expressions were negative.

Treatment modalities

Epithelioid angiosarcoma is considered a high-grade malignancy. The lesion has a sometimes aggressive clinical course [3], and approximately 20% of patients have metastases at the time of initial diagnosis [11]. Therefore, treatment options for angiosarcoma are rather limited. Wide resection with or without chemotherapy and radiation therapy is recommended for solitary angiosarcomas. However, amputation is sometimes necessary for angiosarcomas arising in the extremities. In a previous report, four of 10 cases were treated by amputation [7]. Similar to these previous cases, we performed an above-the-knee amputation due to local recurrence and persistent bleeding.

Penel et al. reported that doxorubicin-based regimens and weekly paclitaxel significantly improved overall survival in patients with metastatic angiosarcoma. The median overall survival in their study was 2.2 months without chemotherapy, 9.7 months with miscellaneous regimens, 11 months with doxorubicin-containing regimens, and 13.1 months with weekly paclitaxel [12]. In a retrospective analysis, Schlemmer et al. also reported that paclitaxel is an effective agent for soft tissue angiosarcoma [13]. To date, there are no reports supporting the efficacy of radiotherapy for angiosarcoma.

Another treatment for angiosarcoma is bisphosphonates. Bone metastases occur in only about 10% of soft tissue sarcomas. In contrast, the incidence of bone metastases is much higher in angiosarcomas, dedifferentiated liposarcomas, and alveolar soft tissue sarcomas, at nearly 50% [14]. Bisphosphonates have proven clinically effective against osteosarcoma [15], chondrosarcoma [16], and Ewing's sarcoma [17]. Vincenzi et al. reported that bisphosphonates significantly prolonged the median time to first bone-related event but did not improve overall survival [18]. Therefore, bisphosphonates are useful in the prevention of pathological fractures when the bone lesions of angiosarcoma are multifocal. Furthermore, bisphosphonates are easier to use in elderly patients and have fewer side effects than chemotherapy.

## Conclusions

We report a rare malignancy, primary epithelioid angiosarcoma of the tibia. Epithelioid angiosarcoma of the bone requires caution as it can be misdiagnosed as metastatic cancer. Vascular endothelial markers such as CD31 and CD34 and epithelial markers such as cytokeratin are useful in differentiating angiosarcoma. As angiosarcomas are sometimes aggressive, amputation was also performed in this case; however, long-term survival was not achieved.
